# Supplementary figures and images for: Immunogenicity and Efficacy of a Recombinant Human Adenovirus Type 5 Vaccine against Zika Virus
Source: Vaccines (Basel). 2020 Apr 7;8(2):170. doi: 10.3390/vaccines8020170 (PMC7349816; doi:10.3390/vaccines8020170)

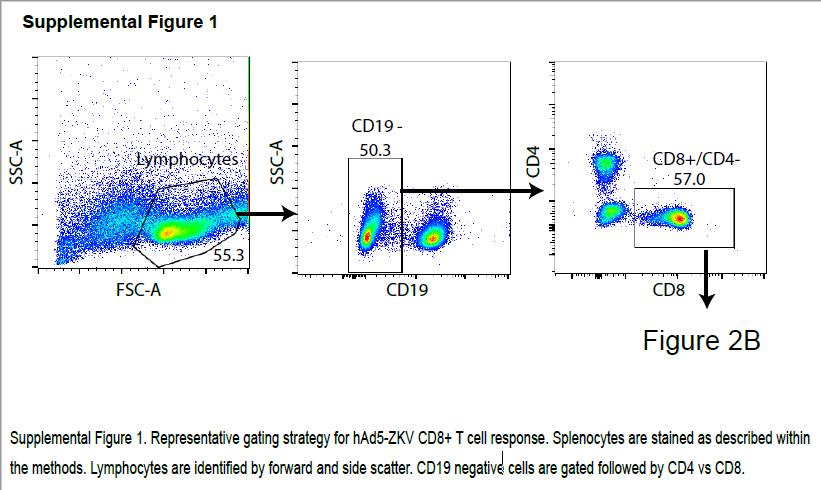

Supplement: Supplementary file 1 [file vaccines-08-00170-s001.zip › vaccines-749266-suppl.png]
